# SD^2^: spatially resolved transcriptomics deconvolution through integration of dropout and spatial information

**DOI:** 10.1093/bioinformatics/btac605

**Published:** 2022-09-05

**Authors:** Haoyang Li, Hanmin Li, Juexiao Zhou, Xin Gao

**Affiliations:** Computational Bioscience Research Center, King Abdullah University of Science and Technology (KAUST), Thuwal 23955, Saudi Arabia; Computer, Electrical and Mathematical Sciences and Engineering Division, King Abdullah University of Science and Technology (KAUST), Thuwal 23955, Saudi Arabia; Computational Bioscience Research Center, King Abdullah University of Science and Technology (KAUST), Thuwal 23955, Saudi Arabia; Computer, Electrical and Mathematical Sciences and Engineering Division, King Abdullah University of Science and Technology (KAUST), Thuwal 23955, Saudi Arabia; Computational Bioscience Research Center, King Abdullah University of Science and Technology (KAUST), Thuwal 23955, Saudi Arabia; Computer, Electrical and Mathematical Sciences and Engineering Division, King Abdullah University of Science and Technology (KAUST), Thuwal 23955, Saudi Arabia; Computational Bioscience Research Center, King Abdullah University of Science and Technology (KAUST), Thuwal 23955, Saudi Arabia; Computer, Electrical and Mathematical Sciences and Engineering Division, King Abdullah University of Science and Technology (KAUST), Thuwal 23955, Saudi Arabia

## Abstract

**Motivation:**

Unveiling the heterogeneity in the tissues is crucial to explore cell–cell interactions and cellular targets of human diseases. Spatial transcriptomics (ST) supplies spatial gene expression profile which has revolutionized our biological understanding, but variations in cell-type proportions of each spot with dozens of cells would confound downstream analysis. Therefore, deconvolution of ST has been an indispensable step and a technical challenge toward the higher-resolution panorama of tissues.

**Results:**

Here, we propose a novel ST deconvolution method called SD^2^ integrating spatial information of ST data and embracing an important characteristic, dropout, which is traditionally considered as an obstruction in single-cell RNA sequencing data (scRNA-seq) analysis. First, we extract the dropout-based genes as informative features from ST and scRNA-seq data by fitting a Michaelis–Menten function. After synthesizing pseudo-ST spots by randomly composing cells from scRNA-seq data, auto-encoder is applied to discover low-dimensional and non-linear representation of the real- and pseudo-ST spots. Next, we create a graph containing embedded profiles as nodes, and edges determined by transcriptional similarity and spatial relationship. Given the graph, a graph convolutional neural network is used to predict the cell-type compositions for real-ST spots. We benchmark the performance of SD^2^ on the simulated seqFISH+ dataset with different resolutions and measurements which show superior performance compared with the state-of-the-art methods. SD^2^ is further validated on three real-world datasets with different ST technologies and demonstrates the capability to localize cell-type composition accurately with quantitative evidence. Finally, ablation study is conducted to verify the contribution of different modules proposed in SD^2^.

**Availability and implementation:**

The SD^2^ is freely available in github (https://github.com/leihouyeung/SD2) and Zenodo (https://doi.org/10.5281/zenodo.7024684).

**Supplementary information:**

Supplementary data are available at *Bioinformatics* online.

## 1 Introduction

Understanding the arrangement of cells and tissues, and its impact on biological function is a fundamental pursuit in life science research (Method of the Year 2020: spatially resolved transcriptomics 2021). Spatially resolved transcriptomics (Ståhl Patrik *et al.*, 2016), which aims to characterize the gene expression profiles while retaining information of spatial tissue context, sheds light on the understanding of structure and function of cells and tissues in recent years (Burgess, 2019). This technique renders the panorama for the organization and heterogeneity of complex tissues by equipping multimodal data containing gene expression profiles with spatial information by capturing the mRNA population of molecules in situ and the super-resolution histological staining image integrating morphological features (Andrews and Hemberg, 2019). Spatial transcriptomics (ST) technique has been utilized for exploring the biological mechanisms among a variety of diseases, tissues and species, such as human heart (Asp *et al.*, 2019), mouse brain (Ortiz *et al.*, 2020), Alzheimer's disease (Chen *et al.*, 2020) and so on.

The cellular composition of biological samples is heterogeneous and varying inherently. Characterizing the variation of cell-type composition across subjects could identify cellular targets of diseases. On the other hand, adjusting for these variations could also clarify the cell–cell interactions and reconstruct the topological and spatial distribution of all cell types (Avila Cobos *et al.*, 2020; Czerwińska, 2018; Dong and Yuan, 2021). The traditional way of bulk sequencing may confound downstream data analysis because less abundant cell types will be masked by that of more abundant ones (Dong *et al.*, 2021; Jin and Liu, 2021). The ability to measure the cellular heterogeneity under specific conditions is therefore critical. However, in the original few generations of ST techniques, the resolution of ST data is much lower than the single-cell level. For instance, the 10× Visium, a commonly used ST technique developed by 10× Genomics, utilizes the spots with 50 µm diameter containing 10–20 cells on average. Thus, unveiling the mixture of cells in the ST spots is a key to depict the precise panorama for the tissues and advance better understanding of the precise tissue organization.

To tackle this problem, several methods have been proposed. SPOTLight (Elosua-Bayes *et al.*, 2021) integrates ST and single-cell RNA sequencing (scRNA-seq) data to infer the cell types of spots in the tissue by seeded non-negative matrix factorization (NMF) regression and non-negative least squares (NNLS) to subsequently deconvolute ST spots. The performance of SPOTLight shows that it could return accurate predictions with shallow sequenced references. DSTG (Su and Song, 2020) utilizes the graph-based model to accurately deconvolute the ST spots and reveal the spatial architecture of cellular heterogeneity in tissues. DSTG has great quantitative performance in benchmarking scRNA-seq of different protocols and identifying cellular heterogeneity in mouse cortex layer, hippocampus slice and pancreatic tumor tissues. Cell2location (Kleshchevnikov *et al.*, 2020), a principled and versatile Bayesian model, integrates the scRNA-seq and ST data to map cell types in situ in a comprehensive manner. The applications on several datasets demonstrate that cell2location could serve as a versatile first-line analysis tool to map tissue architectures. Despite the technical advances, these state-of-the-art methods did not utilize the spatial information among the spots and the non-linear relationship behind various genes. In addition, dropout has been considered as non-informative component by these methods, although both ST and scRNA-seq data show the nature of containing extremely high levels of dropout which may contribute to the deconvolution of ST.

Here, we propose a novel method called SD^2^, which integrates spatial information and embraces an important characteristic, traditionally considered as an obstruction in scRNA-seq analysis, called dropout information, through graph convolutional networks (GCN). In SD^2^, scRNA-seq data with cell-type annotations are complementary resource to generate pseudo-ST data. SD^2^ explicitly utilizes the dropout-based genes of scRNA-seq and ST data, as well as spatial information of all spots as additional information. Non-linear relationship among dropout genes is revealed by auto-encoder (AE) to extract low-dimensional representations of the real- and pseudo-spots. Comprehensive benchmark and real-world experiments demonstrate not only the accuracy of our method over other methods, across various resolutions and measurements, but also the capability to localize the cell types accurately on different ST techniques from mouse brain, mouse kidney and human pancreatic tumor tissues. The utility of dropout information is thus guaranteed as an essential role in the deconvolution task. We further conduct ablation studies to evaluate the importance of each proposed component.

## 2 Materials and methods

### 2.1 Pre-processing

For scRNA-seq data, dropout has been treated as an obstruction to be tackled. However, inspired by Qiu (2020), we leverage dropout as an informative pattern to extract dropout-based genes from ST and scRNA-seq data instead of highly variable genes (HVGs). The input data are ST expression profile $R\in R^{n_{r}\times g_{r}}$ and scRNA-seq expression profile $S\in R^{n_{s}\times g_{s}}\;$where $n_{r}\;(n_{s})$ and $g_{r}\;(g_{s})$ represent the number of spots and genes, respectively. We analyze the distribution of dropout rates in R and S, and find that most of dropout rates are over 80% and some of them are even close to 100% (Supplementary Fig. S1A and B). We identify the dropout-based genes by M3Drop (Andrews and Hemberg, 2019**)**. Through all spots in R and cells in S, a Michaelis–Menten function is fitted to the relationship between mean expression (E) through cells (spots) and dropout-rate ($P_{\mathrm{dropout}}$**)** for each specific gene and the gene-specific parameter $K_{i}$ is estimated by this function. Through the following equation, the global parameter $K_{M}$ is optimized by maximum likelihood estimation across all genes.

(1) Pdropout=1-EE+KM.

The hypothesis is that gene-specific $K_{i}$ is equal to $K_{M}$. After estimating the deviation error from $K_{i}$ to $K_{M}$, significance of each $K_{i}$ is evaluated by *t-test*. Finally, *top-k* significant genes $g_{r}^{d}$ and $g_{s}^{d}$ are chosen for R and S, and we use their expression profiles as the informative features for each spot (cell). The ${g_{d}=g}_{r}^{d}\;\cap g_{s}^{d}$ are intersected genes considered as the preserved features for scRNA-seq and ST data, thus $R{\in R}^{n_{r}\times g_{d}}$ and $S\in R^{n_{s}\times g_{d}}$.

To mimic the gene expressional distribution of real-ST spots better for constructing a homogeneous graph, we generate pseudo-ST spots by annotated scRNA-seq data. Inspired by the concept of Markov Chain Monte Carlo sampling (van Ravenzwaaij *et al.*, 2018), we hope that distribution of more sampled pseudo-ST spots could match the distribution of real-ST spots better. We randomly synthesize m cells as a new pseudo-ST spot whose expression vector would be $\sum_{r}^{m}S_{r}/m$ and the proportion of specific cell-type c is $m_{c}/m$ where $m_{c}$ denotes the number of selected cells in c. The selection of m could refer to the resolution of real-ST spots. For now, pseudo-ST profiles would be $P\in R^{n_{p}\times g_{d}}$, where $n_{p}$ denotes the number of pseudo-ST spots. Then, P and R are divided by the library size through the spots and multiplied by a size factor of 10 000 for normalization. Through generating pseudo-ST spots, the density distribution of counts in pseudo-ST is closer to real-ST than scRNA-seq which means that pseudo-ST generation could mimic the pattern of real-ST better than using individual cells directly (Supplementary Fig. S1C).

To preserve non-linear relationships in the gene expression profiles of all spots, we utilize the AE as the embedding method of the gene expression vector (Dwivedi *et al.*, 2020; Eraslan *et al.*, 2019). We train a three-layer AE network to reconstruct the concatenation of R and P, and minimize the reconstruction error with the encoder ψ and decoder ϕ following Equation 2. Then, we capture the embedded space with dimension $g_{a}$ as the attributes of each spot after the training process. Thus, $R^{\prime }=\psi (R)\in R^{n_{r}\times g_{a}}$ and $P^{\prime }=\psi (P)\in R^{n_{p}\times g_{a}}$, where

(2) ϕ, ψ=argminϕ,ψ MSER, P,ψ ∘ ϕR, P.

### 2.2 Graph construction

Now, we integrate all real- and pseudo-ST spots as nodes into an unweighted graph. For the edges of the graph, we define them at the transcriptional and spatial level. At the transcriptional level, we identify the mutual nearest neighbors between the spots from pseudo-ST and real-ST data. If a pseudo-ST spot $s_{p}$ is among the top-k nearest neighbors of the real-ST spot $s_{r}$ calculated by the *K-Nearest Neighbor (KNN)* algorithm and *vice versa*, we would define that there is a connected unweighted edge between $s_{p}$ and $s_{r}$. k is used to control the sparsity of the graph. This kind of connection preserves the transcriptional similarity between pseudo-ST and real-ST spots. Under the assumption that the expression level among adjacent spots tend to be similar, pseudo-ST data is useful for us to unveil the composition of cell types in the adjacent real-ST spots. At the spatial level, we define the edges among the real-ST spots relying on their spatial coordinates. We set the spot-to-spot horizontal distance as h and the relative coordinates of one specific spot is (x,y), the four nearest neighbors for that spot would be: (x-h/2, y-h/2), (x-h/2, y+h/2), (x+h/2, y+h/2) and (x+h/2, y-h/2). We link these four nearest neighbors to that spot respectively as four unweighted edges and apply this process to all real-ST spots. This spatial connection from real-ST spots could enrich more real-world information for the topological structure of the graph. Finally, the linked graph is

(3) X=P', R'∈RN×ga, N=np+nr

which preserves both spatial and transcriptional structure among all spots, and its adjacency matrix $A\in R^{N\times N}$ would be

(4) Aij=1, if i∼j, 0, otherwise. 

### 2.3 SD^2^

**Fig. 1.**
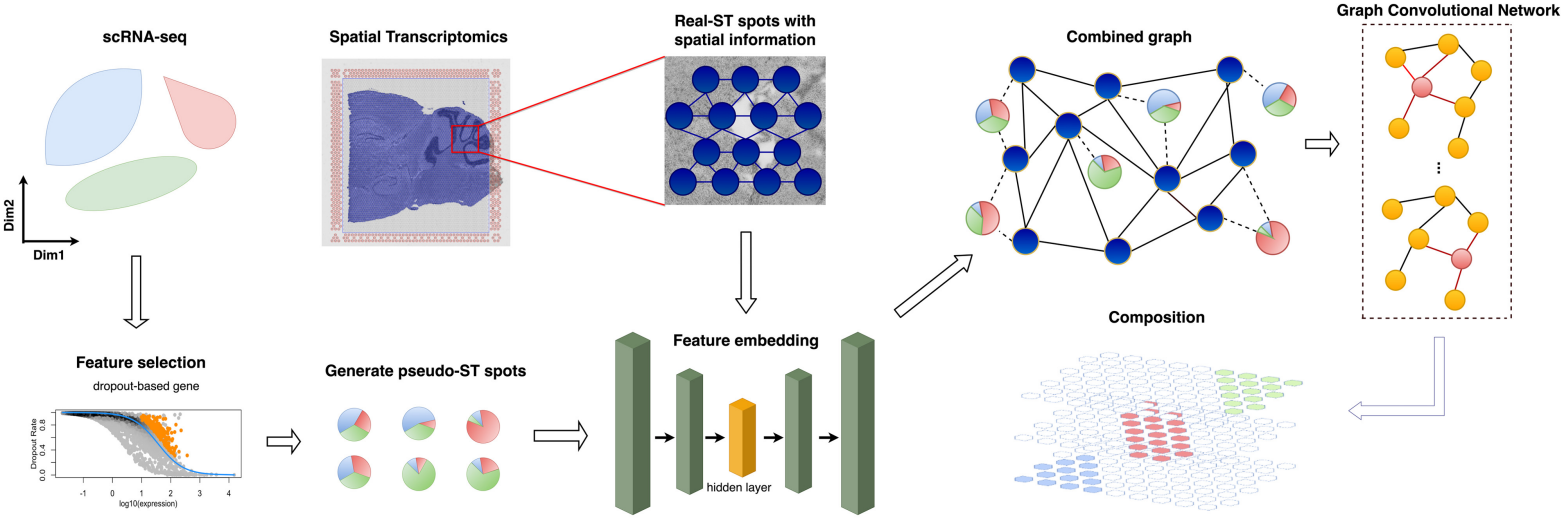
The pipeline of SD^2^. First, we extract the dropout genes by fitting a Michaelis–Menten function and generate the pseudo-ST spots. Then we extract the embedded feature by AE from pseudo-ST and real-ST spots. The pseudo-ST and real-ST spots are constructed as a graph by transcriptional similarity and spatial connection. Finally, graph convolutional neural network is used to output the cell-type composition for real-ST spots

As mentioned above, adjacent spots tend to have similar gene expression patterns. We could unveil the cell-type composition of real-ST spots by utilizing the adjacent pseudo-ST spots with known cell-type composition, which could be formulated as a semi-supervised problem and it comes to GCN as a proper solution. GCN also has the ability for aggregating the graph signals within the node neighborhood which shows capabilities to learn the graph representations and achieves superior performance in a wide range of tasks and applications (Chen *et al.*, 2020). GCN was originally used in the node classification problem (Kipf and Welling, 2017). In that task, the output would contain the probabilities of all classes for each node which would be classified as the class with the highest probability. Since probabilities of all classes for each node are added up to 1 after that softmax activation function, here we treat the classes as cell types and the output probabilities as the composition of all cell types in our case (Fig. 1).

The input data of SD^2^ are expression matrix X and it is adjacency matrix of the constructed graph A. These input data would be fed into GCN with three convolutional layers. To preserve the information of the nodes themselves and train the network more efficiently, the new adjacency matrix is defined as $\tilde{A}={\hat{D}}^{-1/2}\hat{A}{\hat{D}}^{-1/2}$, where $\hat{A}=A+I$ and $\hat{D}$ is the degree matrix of $\hat{A}$. Each layer of GCN could be defined as

(5) Hl+1=fHl,A˜=σA˜HlWl=ReLUA˜HlWl,

where $H^{\left(l\right)}$ is the previous layer, $W^{\left(l\right)}$ is the weight of layer l and we use ReLU as the activation function after each graph convolutional layer. At the output of three convolutional layers, we use softmax as the activation function to normalize the output into the range of $\left[0,1\right]$. Through the network, the cross-entropy is used as the loss function. The output matrix $C=\left[C_{p},\;C_{r}\right]=(c_{tn})\in R^{T\times N}$ represents the cell-type composition matrix of all pseudo-ST and real-ST spots (N spots totally**)** through T cell types and the composition of cell type t in spot n would be $c_{tn}$. The sum of vector $\sum_{t=1}^{T}c_{tn}$ would be 1 in each spot. Thus, final output of SD^2^ would be matrix $C_{r}$.

## 3 Experiments

### 3.1 Experimental setup

During the selection of dropout-based genes, we selected top-2000 significant dropout-based genes and used their expression profiles as the feature for each spot. In the process of generating pseudo-ST spots, we randomly mixed 10–20 cells for each pseudo-ST spots. In AE, we set the dimension of the hidden layer as 200, the batch size as 300 and epoch as 5. In the *KNN* algorithm, we set k as 100. We split the pseudo-ST data into 80% as the training set, 10% as the validation set and 10% as the test set. As SD^2^ is an approach of inductive learning, the training, validation and test set are all utilized in the training process. The validation set is used for preventing overfitting where we set the epoch number of early stopping as 10 and the test set is used for evaluating the performance. Under the grid search technique for hyperparameter optimization, we used Adam optimizer (Kingma and Ba, 2015) with the learning rate as 0.005 among 0.001, 0.005 and 0.01, and training epochs as 200 among 100, 200 and 300. All the experiments were implemented on the 2 Quadro M6000 GPUs in Ubuntu 18.04 operating system.

### 3.2 Benchmark evaluation

For evaluating the performance of SD^2^, we designed quantitative experiments under different resolution of spots and measurements by synthesizing the cells from seqFISH+ dataset (Eng *et al.*, 2019) and MERFISH dataset (Moffitt *et al.*, 2018) to mimic the spots of ST.

**Fig. 2.**
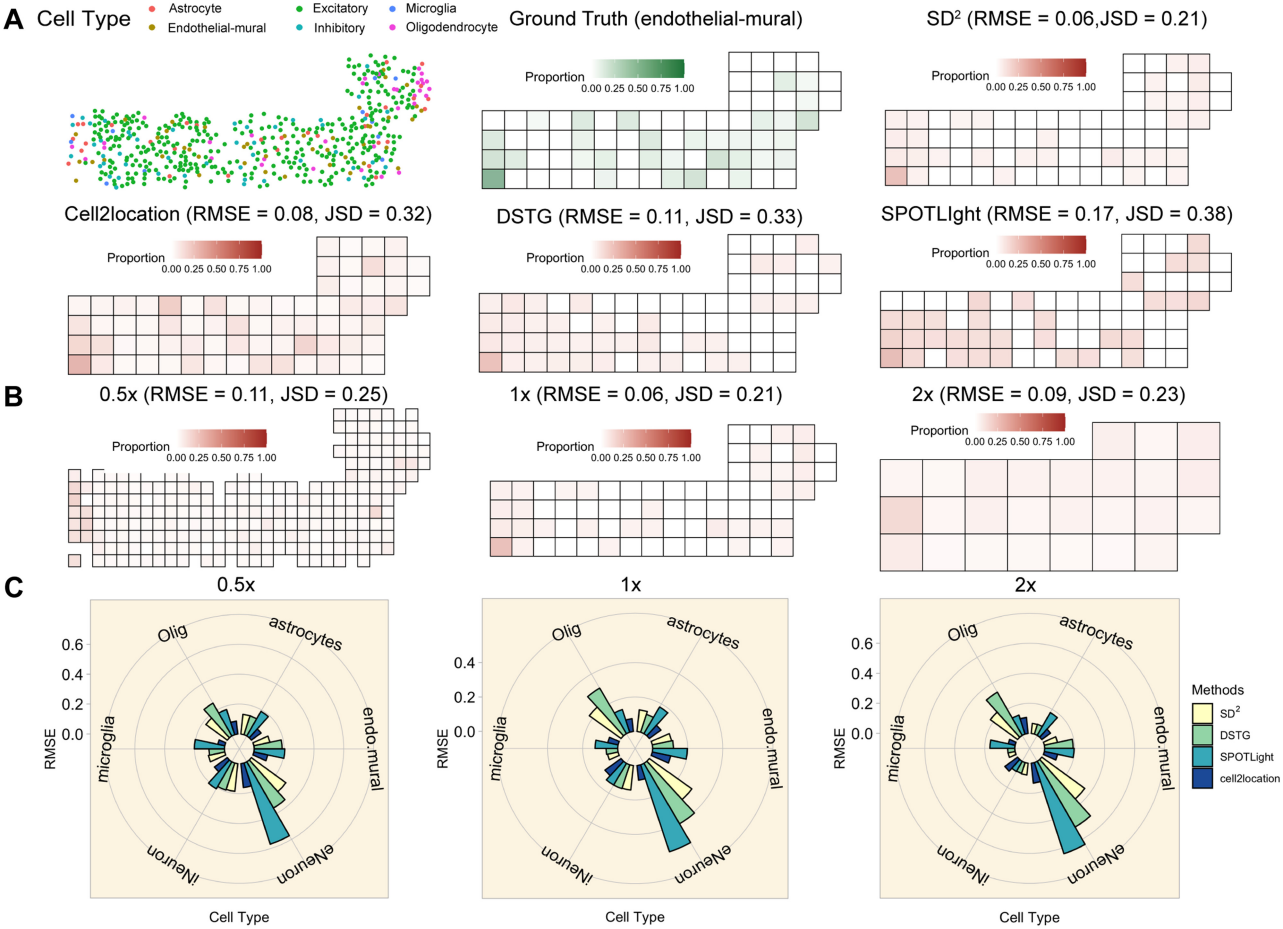
(**A**) The figure at the top left corner shows the spatial and cell-type distribution of the seqFISH+ dataset. The top middle figure shows the ground truth of abundance of endothelial–mural cells in the simulated dataset. The other four figures show the corresponded deconvolution results for endothelial–mural cells from four methods. (**B**) The three spatial distributions of endothelial–mural cells deconvolved by SD^2^ are shown through three kinds of resolution (0.5×, 1× and 2×). (**C**) The three radial column figures compare the RMSE of deconvolution for each cell type on these four methods through three kinds of resolution

seqFISH+ dataset contains 523 cells with corresponding cell-type annotations and spatial coordinates from the cortex of mouse brain at the single-cell resolution. The six cell types of the seqFISH+ dataset are clustered and annotated by the cell type reference of the scRNA-seq dataset (Amit *et al.*, 2015) from the same tissue: excitatory neurons (eNeuron), inhibitory neurons (iNeuron), astrocytes, oligodendrocytes (Olig), microglia cells and endothelial–mural cells (endo_mural) (Fig. 2A). To simulate the low-resolution profiles, we divided these cells by multiple squares and considered one square as a simulated spot with ground truth of cell-type proportions. We designed three side lengths of squares to simulate different resolution of spots [25.75 µm (0.5×), 51.5 μm (1×) and 103 μm (2×)] whose resolutions are 1.2, 4.5 and 15.7 cells in each spot averagely.

To benchmark the performance of SD^2^, we compared with other three methods: SPOTLight (Elosua-Bayes *et al.*, 2021), DSTG (Su and Song, 2020) and cell2location (Kleshchevnikov *et al.*, 2020). We chose to visualize the distribution of proportion of the endothelial–mural cells which has local and distinct pattern of composition among SD^2^ and the other three compared methods. Through the comparison with ground truth, we observed that SD^2^ outperformed the other three methods in root mean square error (RMSE) and Jensen–Shannon Divergence (JSD). In particular, SD^2^ has RMSE of 0.06 and JSD of 0.21 which shows the most similar pattern compared with ground truth (Fig. 2A). We also visualized the proportion of endothelial–mural cells deconvolved by SD^2^ through 0.5×, 1× and 2× resolutions (Fig. 2B). The visualization and corresponding RMSE showed the consistent outperformance over SOTA which demonstrated the robustness of SD^2^. To identify the ability of deconvolution for each cell type, we showed three radial column figures to compare the RMSE of deconvolution for each cell type on these four methods through three kinds of resolution (Fig. 2C). SD^2^ performed better than other three methods for the most cell types and showed the robustness through three kinds of resolutions. We also testified the computational time through these methods and the histogram through the different numbers of genes in ST data (3000, 6000 and 10 000) showed the most efficiency of SD^2^ compared with the other three methods (Supplementary Table S1).

We also evaluated the performance of SD^2^ and three compared methods on the MERFISH datasets of mouse brain medial pre-optic area containing 12 samples from posterior to anterior. MERFISH datasets have 135 genes and 59 651 cells classified by 6 cell types which show the opposite condition of number of genes and spots compared with seqFISH+ dataset, which is helpful to testify the robustness of SD^2^ in these different extreme situations. As the simulation procedure in seqFISH+ dataset, we binned the square of 100 × 100 cells as one spot in MERFISH datasets and 3067 spots were simulated with their real cell-type proportions. We visualized the deconvolution results of SD^2^ and three compared methods in all 12 samples and calculated the average RMSE and JSD of them where SD^2^ still achieved great visualized and quantitative performance compared with the other three methods (Supplementary Fig. S4).

### 3.4 Evaluation on real-world data

To examine the performance of SD^2^ on the real-world data, we collected three datasets: adult mouse brain, mouse kidney and pancreatic ductal adenocarcinoma (PDAC) (Supplementary Table S2).

**Fig. 3.**
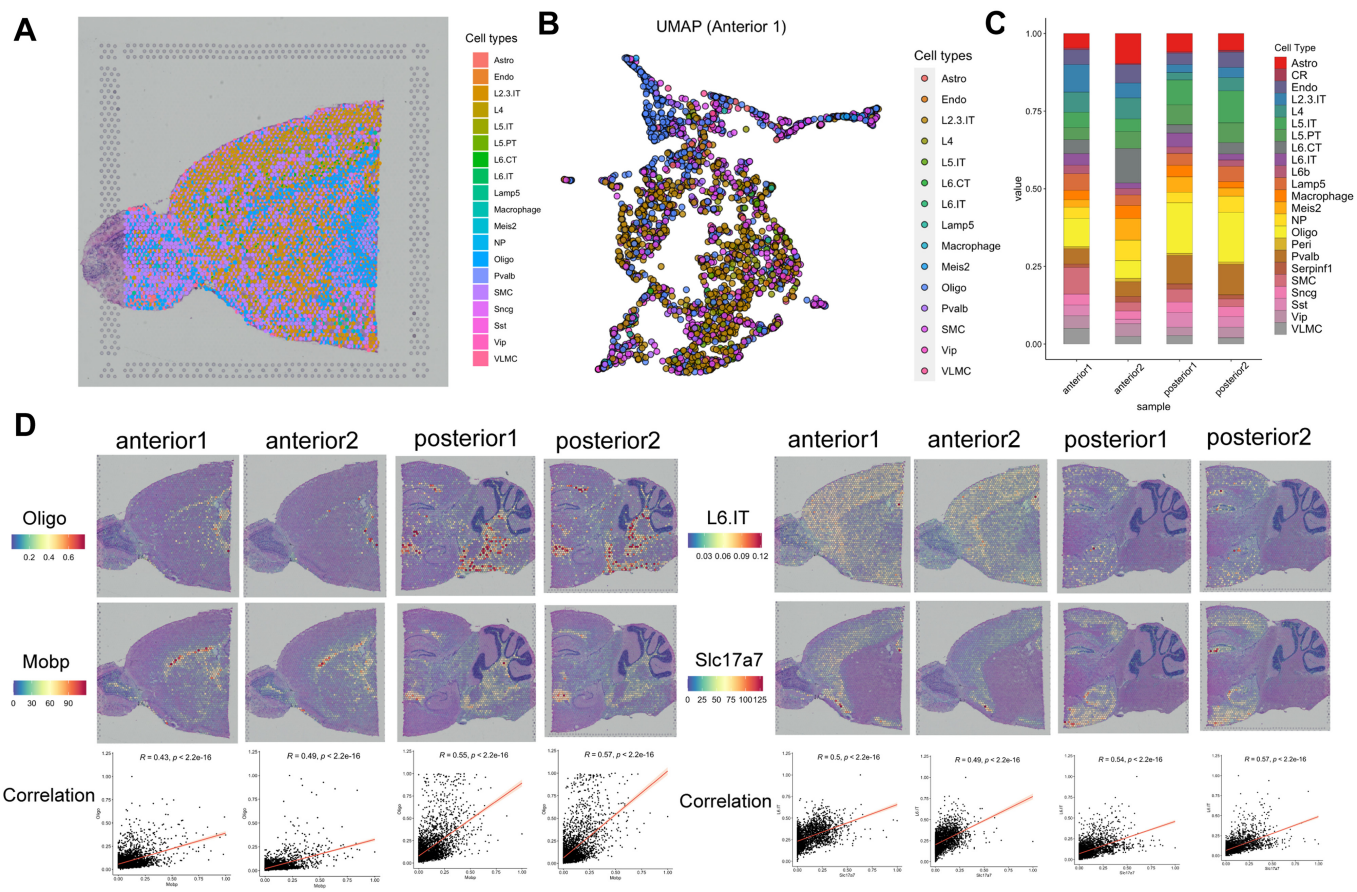
(**A**) The deconvolution results of one spot with all cell types for adult mouse brain. In the figure, each little pie chart indicates a spot with different compositions of cell types noted by different colors. (**B**) UMAP figure of all spots with their highest-content cell type for each spot. Each color represents a specific cell type and all spots are mostly separated following the nature of expression profiles. (**C**) The abundance of all cell types through two anterior slices and two posterior slices. (**D**) Comparison between the abundance of two pairs of cell types and their marker genes: Oligo and Mobp, L6.IT and Slc17a7. The visualized results and Pearson correlation through four slices all show great relationship of them

The cortex of mouse brain is partitioned into multiple subcortical and other cortical regions. The isocortex and hippocampal formation in the mammalian brain could greatly affect the function of perception, cognition, emotion and learning (Rakic, 2009; Van Essen and Glasser, 2018). To explore the heterogeneity of the adult mouse brain, we collected ST data of two anterior and two posterior brain slices from 10× Genomics generated by 10× Visium technique and the scRNA-seq dataset generated by Smart-seq from the Allen Institute (Yao *et al.*, 2021), which consists of around ten thousand cells in adult mouse cortex and hippocampus tissue with 22 cell types. The number of cells could supply various selections for generating the pseudo-ST spots. We also mapped the deconvolution results with spatial coordinates on the original tissue image. After conducting SD^2^, the deconvolution results of four slices from anterior brain and posterior brain were shown (Fig. 3A, Supplementary Fig. S2).

We outputted the cell-type composition of all spots where each little pie chart indicates a spot with different composition of cell types noted by different colors. The cell type whose content in one spot is lower than 5% was deleted from this spot, because the maximum cell number is 20 in a spot by 10× Visium technique, which means that the expected number of such cells is lower than 1. We also visualized the two-dimensional projection of all spots with their highest-abundant cell type by Uniform Manifold Approximation and Projection (UMAP) (Fig. 3B). The UMAP results show that the spots from different colors (cell types) were mostly separated and the spots from the same colors were aggregated together even with the low-resolution of cell-type composition which means that SD^2^ could deconvolve the spots well following the cell-type-specific gene-expression nature. To compare the change of spatial organization of cell type composition quantitatively, we examined abundance of all cell types through four adjacent slices and the smoothness of all abundance of all cell types are shown in Figure 3C which indicated the steady results of SD^2^ through multiple adjacent slices.

We further assumed that the expression pattern of cell-type specific marker genes could reflect to the spatial distribution of cells in that specific cell type, which could also be used to compare with the distribution of the deconvolved cell types by SD^2^. Based on this assumption, we extracted two pairs of cell types and their marker genes: Oligo and Mobp (Holz and Schwab, 1997), and L6 and Slc17a7 (Hodge *et al.*, 2019). Then, we calculated the Pearson correlation and corresponding *P*-value to verify the relationship between cell-type distribution and marker gene’s expression. The results demonstrated the trustiness of SD^2^ that through two anterior and two posterior slices, the Pearson correlation ranged from 0.4 to 0.6 steadily and significantly (Fig. 3D). The visualized results of two pairs of cell-type composition and the distribution of their marker genes’ expression profiles were also matched closely.

We next conducted the experiments on the adult mouse kidney. The kidney maintains fluid, electrolyte and metabolite balance of the body and plays an essential role in blood pressure regulation, red blood cell homeostasis and injury response. It is thus important to understand the cell-type heterogeneity of mouse kidney (Miao *et al.*, 2021; Reidy *et al.*, 2014). We collected scRNA-seq data generated by snATAC-seq from adult mouse kidney including 16 119 cells with 14 cell types (Miao *et al.*, 2021).

**Fig. 4.**
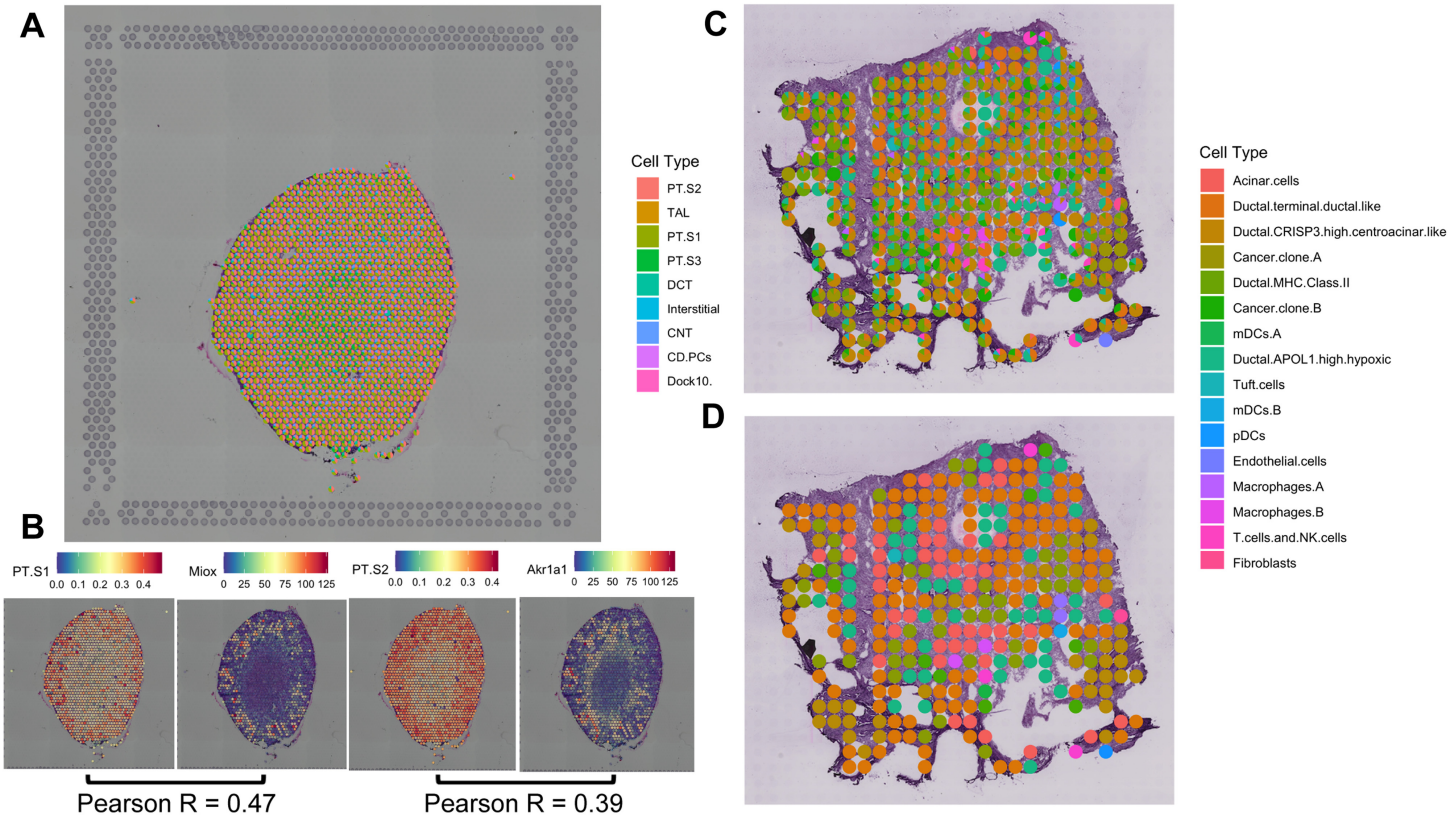
(**A**) The deconvolution results of mouse kidney. (**B**) Comparison between deconvolution results of two cell types (PT S1 and PT S2) with their marker genes’ (Miox and Akr1a1) expression profiles. The Pearson correlation show great relationship between them. (**C**) The deconvolution results of PDAC. (**D**) The low-dimensional deconvolution results where each little pie chart indicates a spot with the highest-content cell type assigned by its specific color

The ST slice of kidney was from 10× Genomics generated by 10× Visium technique whose resolution was 10–20 cells for each spot. We also outputted the cell-type composition of each spot (Fig. 4A) which showed that PT S2 and PT S1 are the top two highest-abundant cell types. We further compared two pairs of abundance of cell types (PT S1 and PT S2) and their specific marker genes’ expression profiles (Miox and Akr1a1) and the results showed great Pearson correlation as 0.47 and 0.39 (Fig. 4B). We then visualized the UMAP results for all spots with its highest-content cell type (Supplementary Fig. S3A). From the visualization of UMAP and the spatial deconvolution results, we could observe that the entropy of cell-type distribution in mouse kidney is higher than that in the mouse brain which means that the cells with the same cell type in mouse kidney are not assembled together.

PDAC is a highly devastating and heterogenic disease for human with poor prognosis and rising incidence (Orth *et al.*, 2019). It is the most prevalent neoplastic disease of the pancreas accounting for more than 90% of all pancreatic malignancies whose 5-year overall survival is less than 8% (Kleeff *et al.*, 2016; Siegel *et al.*, 2018). The ST data of PDAC were generated by the original spatial transcriptomics method (Ståhl Patrik *et al.*, 2016, ) with the low resolution of nearly 10–40 cells for each spot. We collected two ST datasets of PDAC. Paired scRNA-seq data were generated from the pancreatic adenocarcinoma tissue by the InDrop technique (Moncada *et al.*, 2020). PDAC has 1927 cells with 21 cell types. We conducted the experiments on ST data with the corresponding scRNA-seq data and showed the deconvolution results mapping with spatial coordinates and its low-resolution deconvolution results which meant the highest-abundant cell type of each spot was preserved (Fig. 4C and D). We also visualized the UMAP results for all spots with the highest-abundance cell type to observe the data separation among these cell types. Despite the low resolution of these spots, we could still find the relative isolate patterns in UMAP results (Supplementary Fig. S3B). In the results of deconvolution, the cells with the same cell type were aggregated smoothly.

### 3.5 Ablation study

We conducted ablation study to explore the contribution of different modules in SD^2^ on the previous simulated seqFISH+ dataset. We considered four conditions: the original SD^2^; no spatial connection in real ST; no dropout genes; no AE. To replace the dropout-based genes, we used the HVGs as the feature for each spot by analysis of variance. For no spatial connection, we only considered the connection between pseudo-ST and real-ST spots. To replace the AE for embedding features, we used a linear-dimensional reduction method called singular value decomposition and extracted the largest eigenvalues as the final features for nodes. To evaluate the contribution of each module comprehensively, the experiments were conducted in three kinds of resolutions (0.5×, 1× and 2×) and two metrics were used in each experiment: JSD score and RMSE (Table 1). Through the performance of the ablation study, RMSE and the JSD score were both obviously increased through the elimination of our proposed modules which means that each proposed module contributed to the success of SD^2^.

**Table 1.** The ablation study compared the original SD^2^ and the other three conditions: no spatial connection, no dropout-based feature and no AE

Methods	0.5×		1×		2×	
	RMSE	JSD	RMSE	JSD	RMSE	JSD
**SD^2^**	**0.188**	**0.198**	**0.169**	**0.216**	**0.191**	**0.153**
No spatial connection	0.301	0.489	0.181	0.235	0.233	0.255
No dropout-based feature	0.280	0.431	0.188	0.245	0.228	0.237
No AE	0.315	0.482	0.182	0.230	0.238	0.253

*Note*: The experiments were conducted through three resolutions of spot and measured by RMSE and JSD score. The results showed the contribution and necessity of these proposed modules for SD^2^.

To explore the effectiveness of dropout genes, we also designed the experiments on seqFISH+ datasets by three conditions (dropout genes only, HVGs only and both of them) in three numbers of total used genes (3000, 6000 and 10 000 genes) (Supplementary Table S3). The results with different conditions revealed that dropout genes achieved better performance in RMSE and JSD than HVGs steadily in different number of total genes. The combination of dropout genes and HVGs did not show lower RMSE and JSD in most of number of total genes which meant usage of HVGs may be redundant selected features in our datasets.

## 4 Discussion and conclusion

In this article, we proposed a method called SD^2^ for ST deconvolution, which follows the nature of ST to leverage dropout and spatial information in the paired scRNA-seq and ST data. Our comprehensive experiments demonstrate that SD^2^ reaches great quantitative and visible performance under different resolutions, ST techniques and metrics. The comparison with three state-of-the-art methods also shows the superior performance of SD^2^. Based on the transcriptional connection in ST data, we explore the real connection among spots by adding spatial information in ST data under the assumption that adjacent spots tend to have similar gene expression patterns. Under the high dropout rates among ST data, dropout-based shows more utility than highly variable-based on the selection of informative genes and we aim to demonstrate a new perspective that dropout could also play an essential role instead of an obstruction during the analysis of ST data.

For the success of using dropout genes in ST data, it results from several reasons: (i) mRNA expressions in ST are much sparser than scRNA-seq where dropout genes could play a more essential role than HVGs (Supplementary Fig. S1). As the figure shows, the dropout rates of scRNA-seq datasets are around 80% to 90%, but over 90% in ST datasets. The ST datasets has too few counts to select HVGs for deconvolution task. (ii) The selected HVGs could be sensitive to the pre-processing procedure (such as normalization and imputation) which causes the biased selection of HVGs. On the other hand, some informative genes may not be highly variable in the expression profile. (iii) Mathematically, the selection strategy of dropout genes we used assumed that for all genes, dropout rates and mean expression profiles had a non-linear relationship (Michaelis–Menten function) and the outlier genes could be informative to be features. This strategy is only suitable for high dropout rate, such as scRNA-seq and ST data. On the other hand, strategy of HVGs selection could be more suitable for sequencing techniques with high sequence depth, such as RNA-seq. In our study, we validated that dropout genes could be informative features for deconvolution task and they should also be utilized for exploring cell–cell interaction, cell-type clustering or trajectory inference tasks further.

Despite the success of the proposed method, there are still some limitations to be overcome. First, more and more methods begin to use scRNA-seq data to supply more fine-grained transcriptional information. But scRNA-seq and ST data could not be truly gathered from the same tissue sections which means that there must be some inconsistency which affects the deconvolution results for ST data. Second, SD^2^ could be further improved by utilizing the distribution of all scRNA-seq data for assistance. Selecting scRNA-seq data randomly to generate pseudo-ST spots would miss some low-abundance cell types or lose the biological significance. In future work, we would try to solve this issue by narrowing down the scale of selection to generate more realistic spots.

## Funding

This work was supported by the Office of Research Administration (ORA) at King Abdullah University of Science and Technology (KAUST) [BAS/1/1624-01, FCC/1/1976-23-01, URF/1/4077-01-01, URF/1/4098-01-01, REI/1/4216-01-01, REI/1/4437-01-01, REI/1/4473-01-01, URF/1/4352-01-01 and REI/1/4742-01-01].

*Conflict of Interest*: none declared.

## Supplementary Material

btac605_Supplementary_Data

## Data Availability

The seqFISH+ dataset is collected from GSE98674 and corresponded scRNA-seq dataset is collected from GSE6036. The MERFISH datasets are collected from https://datadryad.org/stash/dataset/doi:10.5061/dryad.8t8s248/ and scRNA-seq data is from https://github.com/rdong08/spatialDWLS_dataset/tree/main/datasets. ST and scRNA-seq data from PDAC are collected from GSE111672. ST data from adult mouse brain are collected from 10X Genomics and the corresponding scRNA-seq data are collected from allen institute which are sequenced by smart-seq. The ST data of mouse kidney are collected from 10X Genomics and scRNA-seq data are from GSE157079.
