# Supplementary material for: SD2: spatially resolved transcriptomics deconvolution through integration of dropout and spatial information
Source: Bioinformatics. 2022 Sep 5;38(21):4878–84. doi: 10.1093/bioinformatics/btac605 (PMC9789790; doi:10.1093/bioinformatics/btac605)
Supplement: btac605_Supplementary_Data [file btac605_supplementary_data.pdf]

Supplementary materials of SD^2

Data Availability

The seqFISH+ dataset is collected from GSE98674 and corresponded scRNA-seq dataset is collected from GSE6036. The MERFISH datasets are collected from https://datadryad.org/stash/dataset/doi:10.5061/dryad.8t8s248/ and scRNA-seq data is from https://github.com/rdong08/spatialDWLS_dataset/tree/main/datasets . ST and scRNA-seq data from PDAC are collected from GSE111672. ST data from adult mouse brain are collected from 10X Genomics and the corresponding scRNA-seq data are collected from allen institute which are sequenced by smart-seq. The ST data of mouse kidney are collected from 10X Genomics and scRNA-seq data are from GSE157079.

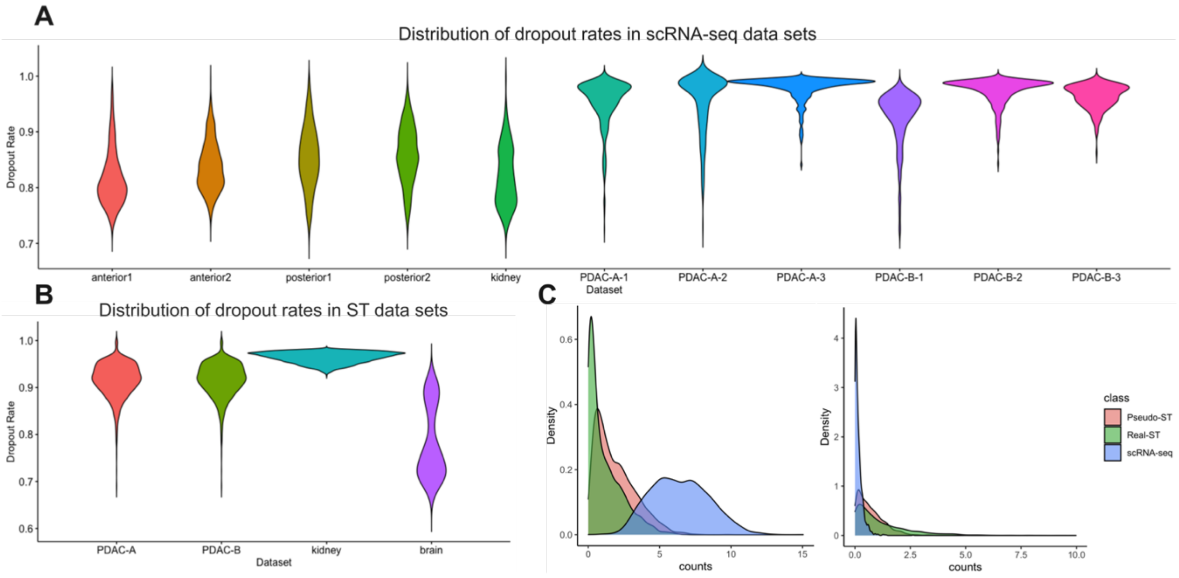

Supp. Fig 1 A. The distribution of dropout rates of all cells in 11 different scRNA-seq data sets. **B.** The distribution of dropout rates of all spots in 4 representative ST data sets. **C.** The density distribution among pseudo-ST, real-ST and scRNA-seq of mouse kidney (left) and mouse brain (right)

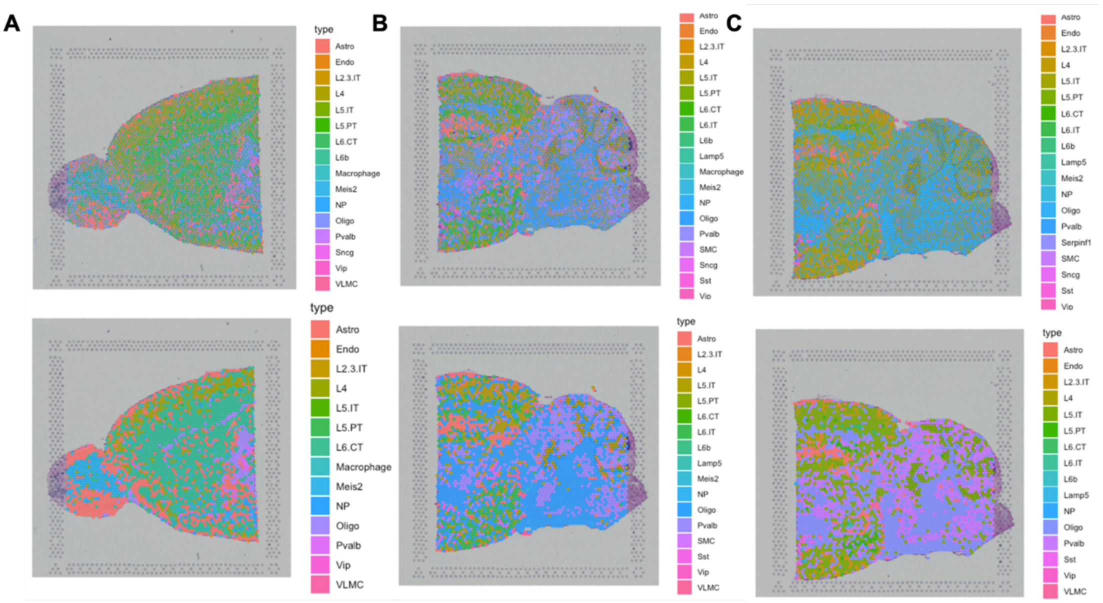

Supp. Fig 2 A. The deconvolution results and low-dimensional result (each spot was represented as the highest-abundance cell type) of second slice of anterior mouse brain. **B.** The deconvolution results and low-dimensional result (each spot was represented as the highest-abundance cell type) of first slice of posterior mouse brain. C. The deconvolution results and low-dimensional result (each spot was represented as the highest-abundance cell type) of second slice of posterior mouse brain.

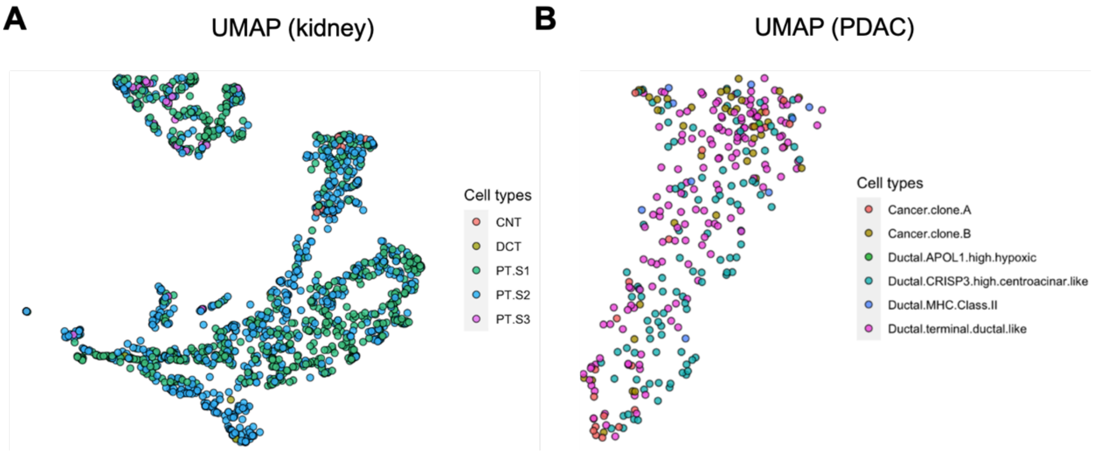

Supp. Fig 3 A. the UMAP results for all spots with the highest-abundant cell type to observe the data separation among these cell types of mouse kidney. **B**. The UMAP results of PDAC.

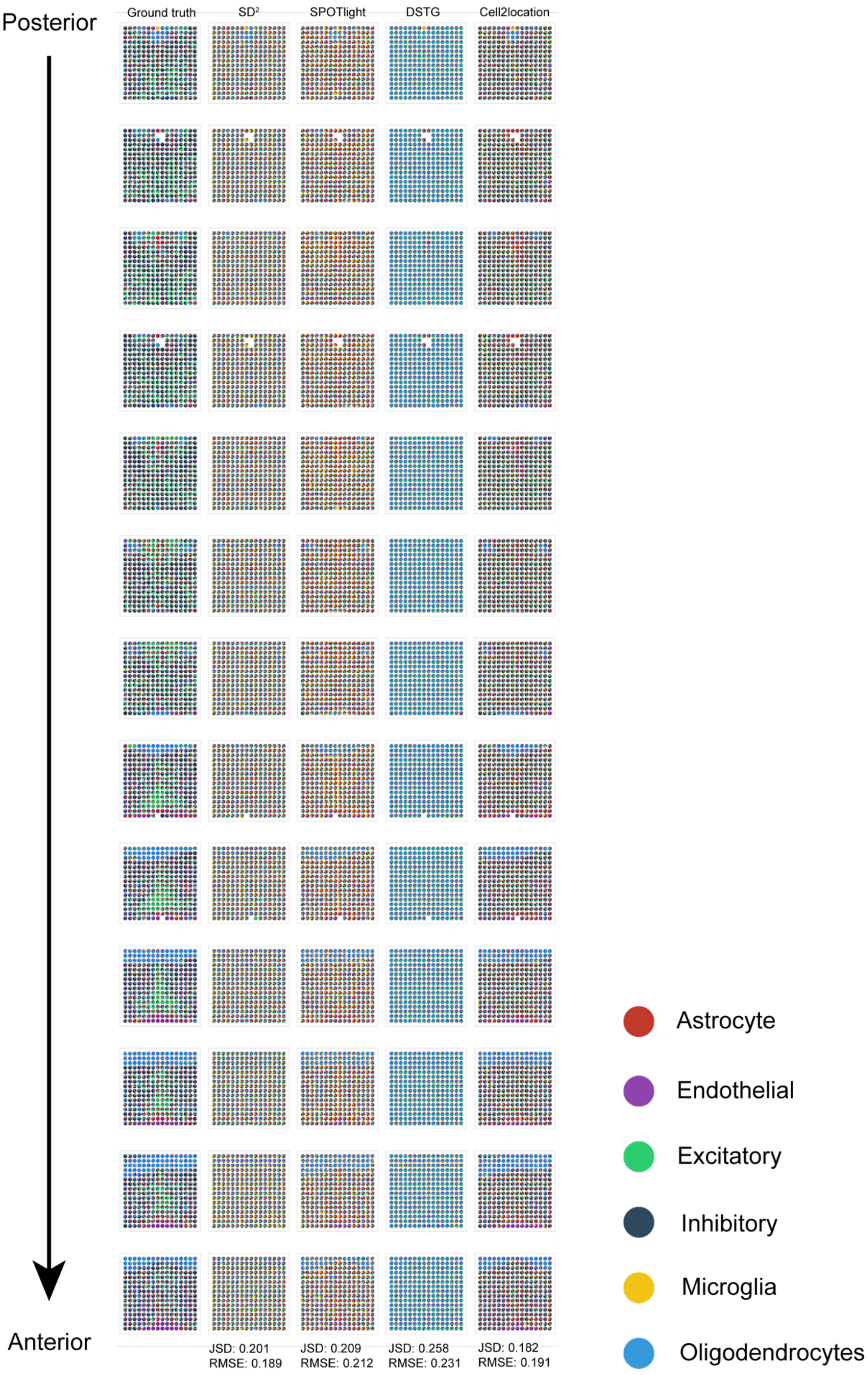

Supp. Fig 4. The visualization results of predicted cell type distribution from four methods and ground truth on MERFISH dataset. The MERFISH dataset contains 12 samples from posterior to anterior. We also calculated the RMSE and JSD score at the bottom of visualization where we could observe that SD2 achieved relatively great performance than the other three methods.

Table 1. Running time (second) of four compared methods through different number of genes in seqFISH+ data.

Number of genes	SD^2	DSTG	SPOTLight	Cell2location
3000	20.5	21.6	134.2	2092.2
6000	27.1	29.2	127.9	1744.4
10000	42.7	40.2	135.3	2023.5

Table 2. Statistics of three real-world data sets.

Tissue	Average ST spots	Cell types of scRNA-seq	Cells of scRNA-seq
Adult mouse brain	3355	22	14249
Adult mouse kidney	1083	14	16119
PDAC	428	21	1927

Table 3. JSD and RMSE for different conditions among only dropout genes, only HVG and both of them through three kinds of gene number on the seqFISH+ dataset.

Gene number	Conditions	JSD	RMSE
	Dropout	0.49223	0.31980
3000	HVG	0.51837	0.33370
	Dropout+HVG	0.52294	0.33358
	Dropout	0.48518	0.32353
6000	HVG	0.55884	0.33701
	Dropout+HVG	0.50494	0.32442
	Dropout	0.52314	0.33187
10000	HVG	0.56464	0.34508
	Dropout+HVG	0.51595	0.32619
